# Prognostic role of MUC5B rs35705950 genotype in patients with idiopathic pulmonary fibrosis (IPF) on antifibrotic treatment

**DOI:** 10.1186/s12931-021-01694-z

**Published:** 2021-04-01

**Authors:** Davide Biondini, Elisabetta Cocconcelli, Nicol Bernardinello, Giulia Lorenzoni, Chiara Rigobello, Sara Lococo, Gioele Castelli, Simonetta Baraldo, Manuel G. Cosio, Dario Gregori, Marina Saetta, Elisabetta Balestro, Paolo Spagnolo

**Affiliations:** 1 Respiratory Disease Unit, Department of Cardiac, Thoracic, Vascular Sciences and Public Health, University of Padova, Via Giustiniani 2, 35128 Padova, Italy; 2 Department of Women’s and Children’s Health, University of Padova, 35128 Padova, Italy; 3 Meakins Christie Laboratories, Respiratory Division, McGill University, Montreal, Canada; 4 Unit of Biostatistics, Epidemiology and Public Health, Department of Cardiac Thoracic Vascular Sciences and Public Health, University of Padova, 35128 Padova, Italy

**Keywords:** Genetics, Idiopathic pulmonary fibrosis, Interstitial lung diseases, *MUC5B*, Polymorphisms

## Abstract

**Background:**

A common variant located in the promoter region of *MUC5B* (rs35705950) is the strongest risk factor for sporadic and familiar IPF, as well as a predictor of outcome. However, there are no data on the effect of *MUC5B* rs35705950 genotype on the prognosis of IPF patients on antifibrotic treatment. The aim of this study is to determine, in a phenotypically well-characterized population of patients with IPF treated with antifibrotics, the impact of *MUC5B* rs35705950 genotype on disease progression and survival.

**Methods:**

88 IPF patients on antifibrotic treatment were followed-up from 2014 until transplantation, death or end of follow-up (December 2019). Disease progression was defined as a forced vital capacity (FVC) loss ≥ 5% per year. All patients were genotyped for *MUC5B* rs35705950 by PCR amplification and Sanger sequencing.

**Results:**

Out of 88 patients, 61 (69%) carried the mutant T allele (TT or TG) and 27 (31%) did not (GG). Carriage of the *MUC5B* rs35705950 T allele was not associated with a faster decline in FVC. Conversely, at the end of the follow-up, overall survival in carriers of the TT/TG genotype was longer compared to that of the GG genotype carriers. FVC (L) at baseline and time to respiratory failure at rest were independent predictors of worse prognosis.

**Conclusions:**

In IPF patients on antifibrotic treatment, carriage of the *MUC5B* rs35705950 T allele is associated with longer survival, highlighting the usefulness of *MUC5B* genetic data in clinical decision making.

**Supplementary Information:**

The online version contains supplementary material available at 10.1186/s12931-021-01694-z.

## Background

Idiopathic pulmonary fibrosis (IPF) is a chronic progressive fibrosing interstitial lung disease of unknown origin, characterized by relentless respiratory failure leading to death within 3–5 years from diagnosis [1]. IPF is believed to occur in genetically susceptible individuals because of an aberrant wound-healing response following repetitive alveolar microinjury, resulting in scarring of the lung parenchyma and irreversible loss of function. IPF is likely to result from a complex interaction between environmental and genetic factors; for instance, as many as 20% of affected individuals report to have a family member with pulmonary fibrosis [2].

In 2011, Seibold and colleagues, using a genome-wide linkage analysis, demonstrated that the minor allele (T) of a single nucleotide polymorphism (SNP) located 3 kb upstream of the *MUC5B* gene transcription start site on 11p15 (rs35705950) was present in 38% of subjects with sporadic IPF and in 34% of subjects with familial interstitial pneumonia [3]. Notably, the risk of disease development increased in a dose-dependent manner, from an odds ratio of 9 for heterozygous carriers of the T allele (i.e., GT) up to 21.8 for the homozygous carriers [3]. The association of *MUC5B* rs35705950 with IPF has been replicated in several independent cohorts [4–8] and represents the strongest genetic risk factor for sporadic and familial IPF described thus far.

*MUC5B* encodes a mucin 5B precursor protein that contributes to airway mucus production and homeostasis [9]. Although the precise mechanisms through which *MUC5B* dysregulation contributes to IPF development are currently unknown, *MUC5B* overexpression may cause mucociliary dysfunction, retention of particles and disruption of the normal reparative mechanisms in the distal lung, leading to chronic fibroproliferation and regenerative process that results in honeycomb cyst formation [10–14].

*MUC5B* rs35705950 T allele not only predisposes to IPF but has also been associated with improved survival, although this latter association remains debated and somehow controversial.

With this background, the aim of our study was to evaluate the influence of *MUC5B* rs35705950 genotype on disease behavior and survival of IPF patients on antifibrotic treatment. To the best of our knowledge, this has never been investigated before.

## Methods

### Study population and study design

In this longitudinal retrospective study, we analyzed a consecutively collected cohort of well-characterized Caucasian adult patients with sporadic IPF referred to our center between April 2014 and September 2018. Patients were followed-up until transplantation, death or end of follow-up (December 2019), and those who permanently discontinued treatment were excluded from the study. Eighty-eight patients were included in the study (Table 1). The diagnosis of IPF was re-evaluated according to the ATS/ERS/JRS/ALAT guidelines [1]. Occupational or environmental exposure and connective tissue disease were excluded, and only sporadic IPF were considered for the analysis.

**Table 1:** Clinical and functional characteristics of the entire IPF population, IPF patients with TT/TG genotype and with GG genotype

	Entire population (n = 88)	TT/TG genotype (n = 61)	GG genotype (n = 27)	*p* Value
Male, n (%)	71 (81)	49 (80)	22 (81)	0.99
Age at diagnosis, years	70 (44–84)	69 (44–84)	71(50–82)	0.30
Body mass index, kg/m^2^	26 (19–37)	26 (19–33)	27 (22–37)	0.49
Smoking history, pack years	10 (0–240)	10 (0–50)	30 (0–240)	**0.0001**
Current, n (%)	7 (8)	5 (8)	2 (7)	
Former, n (%)	59 (67)	38 (62)	21 (78)	0.31
Nonsmokers, n (%)	22 (25)	18 (30)	4 (15)	
Radiological diagnosis, n (%)	49 (56)	29 (48)	20 (74)	**0.03**
UIP	49	29	20	
Probable UIP	31	24	7	**0.03**
Indeterminate UIP	8	8	0	
FVC at baseline, L	2.60 (1.20–4.61)	2.68 (1.56–4.36)	2.32 (1.20–4.61)	**0.02**
FVC at baseline, %pred	77 (47–126)	78 (52–126)	68 (47–118)	**0.05**
TLC at baseline, %pred	73 (40–96)	73 (45–96)	73 (40–93)	0.37
DL_CO_ at baseline, %pred	56 (7–93)	56 (7–89)	56 (28–93)	0.67
Gastroesophageal reflux, n (%)	32 (36)	23 (38)	9 (33)	0.69
Cardiovascular diseases, n (%)	63 (72)	44 (72)	19 (70)	0.86
Metabolic syndrome, n (%)	37 (42)	25 (41)	12 (44)	0.76
Pirfenidone treatment, n (%)	51 (58)	37 (61)	14 (52)	0.48
Nintedanib treatment, n (%)	37 (42)	24 (39)	13 (48)	0.48
FVC decline in the 1^st^ year– mL	50 (-573–657)	84 (-573–657)	34 (-559–461)	0.54
FVC decline in the 1^st^ year, %pred	1 (-29–21)	1 (-29–21)	0 (-12–16)	0.80
Stable in the 1st year, n (%)	63 (72)	45 (74)	18 (67)	0.60
Progressors in the 1st year, n (%)	25 (28)	16 (26)	9 (33)	
RF on exercise, months	19 (0–89)	21 (0–89)	16 (0–44)	0.13
RF at rest, months	27 (0–110)	31 (5–110)	24 (0–59)	**0.04**
Nausea or vomiting, n (%)	15 (17)	13 (21)	2 (7)	0.10
Diarrhea, n (%)	16 (18)	12 (20)	4 (15)	0.58
Weight loss, n (%)	25 (28)	19 (31)	6 (22)	0.39
Increase in AST, ALT, n (%)	2 (2)	2 (3)	0 (0)	0.34
Acute exacerbations	5 (6)	3 (5)	2 (7)	0.56
Lung transplant, n (%)	5 (6)	4 (6)	1 (4)	0.17
Death, n (%)	27 (31)	15 (25)	12 (44)	0.06

*FVC*  forced vital capacity, *TLC* total lung capacity, *DLCO* lung diffusion carbon oxide, *RF* respiratory failure, *AST* aspartate aminotransferase, *ALT* alanine aminostransferase. Values are expressed as numbers and (%) or median and ranges as appropriate. Negative values mean improvement of FVC. To compare demographic data and baseline clinical characteristics between TT/GT genotype and GG genotype, Chi square test and Fisher t test (n < 5) for categorical variables and Mann–Whitney U test for continuous variables were used. *P*-values < 0.05 were considered statistically significant (bold values)

Patients were followed clinically and functionally for at least one year after initiation of antifibrotic. Patients were treated with pirfenidone or nintedanib according to eligibility criteria and the risk of associated adverse events.

Based on their annual rate of decline in absolute FVC% pred. during the first year of treatment, patients were defined as progressors (≥ 5%pred.) or stable (< 5%pred.), as previously reported [15, 16]. Improvement of FVC was expressed as negative value.

The progression-free survival (PFS) was calculated from the time of treatment initiation until functional progression, which was defined as absolute FVC% pred. loss ≥ 5% compared to the basal FVC% pred.

Based on the level of oxygen in the blood (PaO2), we defined respiratory failure when this value was < 60 mmHg (8.0 kPa).

The time to development of respiratory failure (RF) on exercise and at rest was defined as the time from treatment initiation and development of RF.

The occurrence of acute exacerbation of IPF, defined as an acute worsening of dyspnea with bilateral ground glass opacities superimposed on the UIP pattern not fully explained by fluid overload [17], has been collected.

Blood sample was taken for each patient included in the study for DNA extraction and *MUC5B* rs35705950 genotyping. Based on their *MUC5B* genotype, patients were then divided in two groups (TT/TG or GG genotype).

The study was performed in accordance with the Declaration of Helsinki and was approved by the Ethics Committee of the University Hospital of Padova (4280/AO/17). Informed consent was obtained for all study participants.

Sample processing were described in the Additional file 1.

### Statistical analysis

Categorical variables are described as absolute (n) and relative values (%) whereas continuous variables are described as median and interquartile range. To compare demographic data and baseline clinical characteristics between TT/TG and GG genotypes, Chi square test and Fisher’s exact test for categorical variables and Mann–Whitney U test for continuous variables were used, as appropriate. Due to the low number of events, in survival analysis, death and death/lung transplantation were combined. Survival was estimated using the Kaplan–Meier method and the p-value of the log-rank test was reported. Analysis on progression was conducted using the Cumulative Incidence Functions (CIF) to account for competing risks.

Clinical characteristics were evaluated to determine their relationship with survival in a univariate analysis of Cox proportional hazards regression testing. The time dependency was evaluated via visual examination of Schoenfeld residuals plot. Variables with a statistically significant association with overall survival on univariate analysis were included in a multivariate Cox proportional hazard regression test to find factors independently associated with disease progression.

All data were analyzed using SPSS Software version 25.0 (New York, NY, US: IBM Corp. USA) and R software. P-values < 0.05 were considered statistically significant.

## Results

### Clinical and functional characteristics

Clinical and functional characteristics at baseline for the entire study population are shown in Table 1. Most patients were males and former smokers with a median age at diagnosis of 70 years. Almost half of the patients (56%) had a radiological diagnosis, while the remaining required histological confirmation. Mean FVC was 77%, reflecting a mild functional defect, and cardiovascular disease represented the most frequent comorbidity (72%). During their first year of treatment, 63 patients (72%) remained functionally stable while 25 (28%) progressed. Over the entire study period (2014–2019) 27 patients (31%) died, 5 (6%) were transplanted and 5 (6%) experienced an acute exacerbation.

The allele frequency of the *MUC5B* rs35705950 T was 42% (74/176), while the frequency of the wild type G allele was 58% (102/176). The MUC5B rs35705950 genotype frequencies met the Hardy–Weinberg equilibrium (Additional file 2: Table S1).

Based on the absence or presence of the minor allele (T) either in homozygosity or in heterozygosity, the population was categorized in two groups: patients with TT/TG genotype (n = 61, 69%) and with GG genotype (n = 27, 31%) (Table 1). The two groups did not differ regarding age, sex, body mass index, comorbidities and antifibrotic treatment. Patients carrying the GG genotype had consistently higher smoking history (30 vs. 10 PY; p < 0.001), lower FVC at treatment start (2.32 vs. 2.86L, p = 0.02; 68 vs. 78%, p = 0.05) and more radiological diagnosis (74 vs. 48%, p = 0.03) compared to TT/TG genotype. However, FVC decline (at the first year) and the percentage of patients with stable disease were similar between the two groups. Respiratory failure (RF) at rest occurred later in patients with the TT/TG genotype (31 vs. 24 months, p = 0.04) (Table 1).

### Progression-free survival and survival analysis

**Fig. 1:**
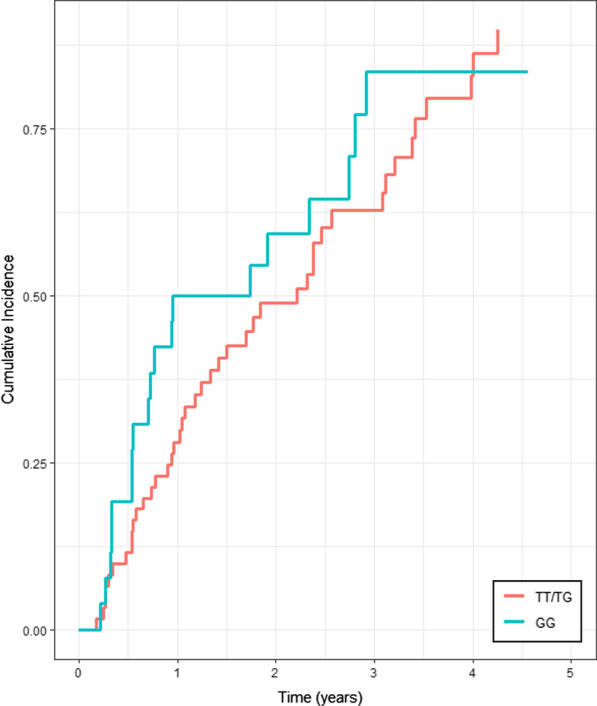
Progression-free survival of TT/TG and GG genotype patients. The red line represents the progression-free survival in the TT/TG group and the green line represents the progression-free survival in the GG group. Kaplan Meier analysis was used with a log-rank test (HR 1.41, 95% CI 0.81–2.44; p = 0.21)

**Fig. 2:**
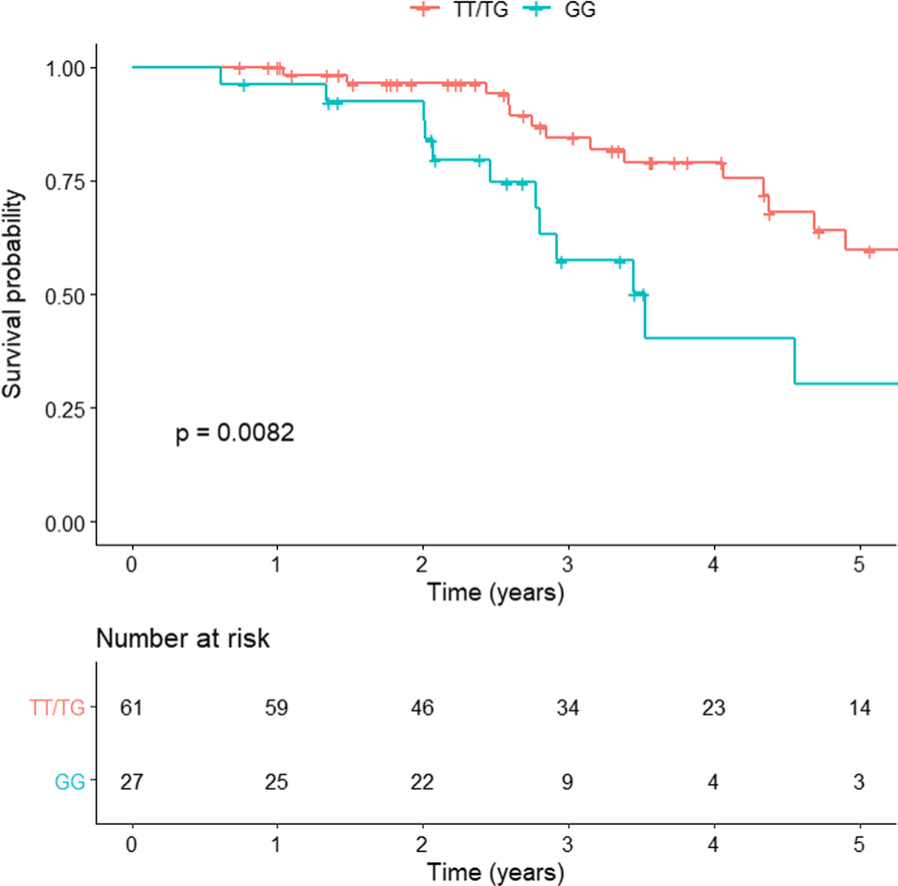
Survival analysis of TT/TG and GG genotype patients. The red line represents the survival in the TT/TG group and the green line represents the survival in the GG group. Kaplan Meier analysis was used with a log-rank test (HR 2.59, 95% CI 1.24–5.40; p = 0.0082)

The progression-free survival was similar between patients with the TT/TG and GG genotypes, with a median of 19 months and 20 months, respectively (p = 0.21) (Fig. 1). On univariate analysis earlier occurrence of RF at rest and on exercise and higher levels of neutrophils were associated with disease progression. However, on multivariate analysis, only earlier occurrence of RF at rest (HR 2.36, 95%CI 1.12–4.97; p = 0.02) was independently associated with disease progression in the entire population (Additional file 3: Table S2). Conversely, *survival analysis* revealed that patients carrying the GG genotype had a significantly worse survival than patients carrying the TT/TG genotypes (42 vs. 74 months, respectively; HR 2.59, 95%CI 1.24–5.40, p = 0.0082) (Fig. 2).

On multivariate analysis, earlier occurrence of RF at rest (HR 36.7, 95%CI 2.83–47.78; p = 0.006) and lower FVC (L) at treatment initiation (HR 77.2, 95%CI 2.99–199.0; p = 0.009) were significantly associated with mortality (Table 2).

**Table 2:** Predictive factors of overall mortality in the entire population of IPF patients treated with antifibrotics

		Univariate analysis		Multivariate analysis	
		HR (95% CI)	*p* Value	HR (95% CI)	*p* Value
Sex	Female	–	–	–	–
	Male	1.33 (0.51–3.49)	0.55	–	–
Age at diagnosis (years)	< 70	–	–	–	–
	≥ 70	1.26 (0.61–2.58)	0.52	–	–
BMI (kg/m^2^)	< 26	–	–	–	–
	≥ 26	0.85 (0.41–1.73)	0.66	–	–
Smoking history (packyears)	< 10	–	–	–	–
	≥ 10	1.72 (0.83–3.59)	0.14	–	–
Smoking status	No	–	–	–	–
	Current	1.91 (0.36–10.01)	0.44	–	–
	Former	1.93 (0.76–4.88)	0.16	–	–
Gastroesophageal reflux	No	–	**–**	–	–
	Yes	0.26 (0.11–0.64)	**0.003**	0.11 (0.09–1.6)	0.1
Cardiovascular diseases	No	–	–	–	–
	Yes	1.57 (0.69–3.56)	0.27	–	–
Metabolic syndrome	No	–	–	–	–
	Yes	0.90 (0.42–1.92)	0.79	–	–
Treatment type	Nintedanib	–		–	–
	Pirfenidone	2.27 (0.78–6.60)	0.13	–	–
MUC5B rs35705950	TT/TG	–	**–**	–	–
	GG	2.39 (1.12–5.06)	**0.02**	1.75 (0.09–31.8)	0.7
Respiratory failure at rest (months)	≥ 26	–	**–**	–	–
	< 26	9.44 (4.10–21.77)	** < 0.0001**	36.7 (2.83–47.7)	**0.006**
Respiratory failure on effort (months)	≥ 19	–	**–**	–	–
	< 19	4.54 (2.06–10.00)	** < 0.0001**	4.96 (0.45–53.8)	0.18
Nausea and vomiting during treatment	No	–	–	–	–
	Yes	0.64 (0.24–1.68)	0.37	–	–
Weight loss during treatment (Kg)	No	–	–	–	–
	Yes	0.96 (0.39–2.34)	0.93	–	–
Diarrhea during treatment	No	–	**–**	–	–
	yes	0.17 (0.04–0.74)	**0.02**	0.45 (0.04–4.73)	0.5
Increase in AST and ALT	No	–	–	–	–
	Yes	6.42 (0.78–52.41)	0.08	–	–
FVC at treatment initiation (L)	≥ 2.60	–	**–**	–	**–**
	< 2.60	3.03 (1.42–6.48)	**0.004**	77.2 (2.99–199.0)	**0.009**
FVC at treatment initiation (%)	≥ 77	–	–	–	–
	< 77	1.80 (0.87–3.71)	0.11	–	–
TLC at treatment initiation (%)	≥ 73				
	< 73	1.89 (0.90–3.74)	0.09		
DL_CO_ at treatment initiation (%)	≥ 56	–	–	–	–
	< 56	1.30 (0.64–2.65)	0.45	–	–
FVC after 1-yr of antifibrotic drug (L)	≥ 2.56	–	**–**	–	–
	< 2.56	2.25 (1.08–4.94)	**0.04**	0.16 (0.01–2.21)	0.17
FVC decline in 1-yr of antifibrotic drug (ml)	< 50	–	–	–	–
	≥ 50)	1.13 (0.52–2.47)	0.74	–	–
FVC after 1-yr of antifibrotic drug (%)	≥ 78	–	**–**	–	–
	< 78)	2.61 (1.10–6.19)	**0.03**	0.68 (0.10–4.23)	0.68
FVC decline in 1-yr of antifibrotic drug (%)	< 1.02	–	–	–	–
	≥ 1.02	1.44 (0.67–3.12)	0.34	–	–
Disease progression	Stables	–	–	–	–
	Progressors	2.12 (0.90–4.98)	0.08	–	–
TLC after 1-yr of antifibrotic drug (%)	≥ 69	–	**–**	–	–
	< 69	2.30 (1.04–5.08)	**0.04**	7.07 (0.95–52.66)	0.56
TLC decline in 1-yr of antifibrotic drug (%)	< 3.02	–	–	–	–
	≥ 3.02	1.96 (0.85–4.49)	0.11	–	–
DLCO after 1-yr of antifibrotic drug (%)	≥ 54	–	–	–	–
	< 54	1.47 (0.67–3.21)	0.33	–	–
DLCO decline in 1-yr of antifibrotic drug (%)	< 0	–	–	–	–
	≥ 0	1.52 (0.69–3.35)	0.3	–	–

*FVC* forced vital capacity, *TLC*  total lung capacity, *DLCO*  lung diffusion carbon oxide, *RF*  respiratory failure, *AST*  aspartate aminotransferase; *ALT*  alanine aminostransferase. Values are expressed as HR (95%CI). Univariate and multivariate Cox proportional hazard regression tests were used to determine the relationship of clinical, functional and radiological characteristics with progression. *P*-values < 0.05 were considered statistically significant (bold values)

**Fig. 3:**
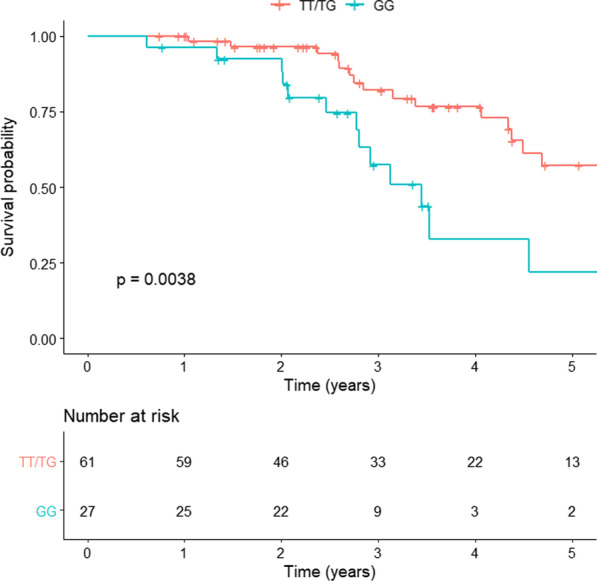
Combined survival and transplantation analysis of TT/TG and GG genotype patients. The red line represents the analysis in the TT/TG group and the green line represents the analysis in the GG group. Kaplan Meier analysis was used with a log-rank test (HR 2.73, 95% CI 1.34–5.54; p = 0.0038)

When death is considered together with transplantation, we confirmed that patients carrying the GG genotype had a significantly worse survival than patients carrying the TT/TG genotypes (41 vs. 71 months, respectively; HR 2.73, 95%CI 1.34–5.54, p = 0.0038) (Fig. 3).

In further analysis, stratifying patients with the TT/TG and GG genotypes by the median time to RF at rest (26 months) and FVC at treatment start (2.6L), a significantly higher percentage of GG genotype carriers had a FVC lower than the median value (67 vs. 41%, p = 0.02), whereas no differences were observed with regard to development of RF at rest (Additional file 4: Table S3).

## Discussion

This study shows for the first time that in IPF patients on antifibrotic treatment, survival may be affected by carriage of *MUC5B* rs35705950 T allele, whether in homozygous or heterozygous form.

MUC5B encodes a major gel-forming mucin that is secreted by proximal submucosal glands and distal airway secretory cells, and plays a key role in mucociliary clearance and host defense [10–14]. A common variant in the promoter region of *MUC5B* gene has been identified as the strongest genetic risk factor for sporadic and familiar pulmonary fibrosis, although its role in disease development remains speculative. Moreover, mutant T allele has also been associated with pulmonary fibrosis in asbestosis [18], chronic HP [19] and rheumatoid arthritis-ILD [20].

Whether carriage of the mutant rs35705950 T allele has prognostic implications in patients with IPF is also debated, and conflicting results have been reported. However, these studies were performed before antifibrotics became the standard of care for patients with IPF and the effect of *MUC5B* rs35705950T on treatment response could not be assessed.

The finding of our study is in line with previous work by Peljto et al. [6], who described the protective effect of the *MUC5B* rs35705950 T allele in two IPF independent cohorts, one enrolled in the INSPIRE trial and the other recruited at the University of Chicago between 2007 and 2010. Moreover, rs35705950 T was also reported to be independently associated with lower bacterial burden in the bronchoalveolar lavage (p = 0.01), lower lung function decline and mortality [21].

Conversely, Jiang and colleagues showed that T allele was associated to increased mortality in a Chinese population [22]; specifically, T allele carriers had a more severe disease, as assessed by lower FVC and DL_CO_. One bias that makes it difficult to compare these studies was the T allele frequency of 20%, consistently lower to that reported in previous studies [3, 6, 7] (almost 40%) and replicated in our cohort. Indeed, as with many other genes, the frequency of *MUC5B* polymorphisms depends on the individual’s ethnic background, with a lower prevalence reported among Asians compared to white non-Hispanics [23].

Nonetheless, the prognostic role of MUC5B polymorphism is under debate, and conflicting results have been recently published as abstracts by two study groups, where no effect of MUC5B variant on survival in IPF patients has been shown [24, 25]. In both cases, it was not clarified whether IPF patients were on antifibrotic treatment or not.

The reason why the T allele may increase the risk of developing IPF in the general population, but confers a survival advantage within the IPF population, can only be speculated upon.

The genetic peculiarity of the *MUC5B* rs35705950 polymorphism resides in being a common variant with a high effect. Indeed, variants that are common in the general population (i.e., polymorphisms) rarely determine significant clinical or biological effects, except for conferring increased disease susceptibility. Conversely, rare variants (i.e., mutations) tend to be highly penetrant with substantial phenotypic effect. The wild type (G) and mutant (T) allele may interact with distinct environmental factor to determine opposite effect on disease susceptibility and prognosis, but this needs to be explored further. Intuitively, carriers of the T allele may have a better survival than noncarriers as a result of a slower disease progression, but this does not seem to be the case. Indeed, evidence of an association of less severe pathological changes and *MUC5B* polymorphism is reported, but it is not clear how these changes were defined [26]. Moreover, in a study by Stock [7], it was described only a trend towards a longer time to decline in FVC (HR 0.59, p = 0.052) in those carrying the T allele when multivariate stepwise regression was used.

IPF population in our cohort had a relatively stable disease under antifibrotics, with FVC decline of approximately 50 mL/year, similar in TT/TG and GG carriers. Moreover, the survival rate was very high, up to 70% at 5 years with only 5 cases of acute exacerbations leading the patient to death, confirming the efficacy of antifibrotic treatment in reducing mortality and also acute exacerbations. This rate is higher to that reported in literature; indeed, a recent study described survival rate of the INSIGHT-IPF registry [27] of nearly 60% at 2 years in the treated group, but the disease was more severe compared to our cohort.

Similarly to FVC decline, no between-group difference was observed in progression-free survival, that was nearly two years, supporting the beneficial effect of antifibrotic treatment in IPF, irrespective of *MUC5B* genotype [28–30].

However, at treatment initiation the two groups differed in terms of FVC, which was an independent predictor of mortality. Functional differences between TT and GG genotypes were described also by Peljto and coworkers [6], but authors did not clarify whether the difference was significant; however, in multivariate analysis, *MUC5B* genotype was associated with survival independently from FVC.

Given the prognostic role of FVC in IPF, it is not surprising that patients with a lower FVC at baseline had a worse survival, and that patients with more preserved lung function at diagnosis live longer [31, 32]. What remains difficult to explain, and somehow counterintuitive, is why patients with more preserved lung function are diagnosed earlier. Answering to this question requires larger prospective studies.

MUC5B has an important role in airway immunity, similar to other mucins, by capturing and removing infectious agents through mucociliary clearance [33]. *MUC5B* rs35705950 T allele is associated with overproduction and accumulation of mucin in distal airspaces and this could lead to an impaired mucociliary activity, that may trigger cough [3]. Interestingly, the mutant MUC5B allele has also been associated with cough severity [34]. Therefore, patients with early cough may seek medical attention when their lung function is still preserved, which may confer a survival benefit.

Another potential consequence of mucociliary dysfunction is the retention of inhaled substances (air pollutants, cigarette smoke, microorganisms, etc.) and endogenous inflammatory debris that over time may result in temporally and spatially distinct areas of microscopic scaring and progressive fibroproliferation in the lung. In this regard, Seibold [3] reported an association between *MUC5B* gene polymorphism and honeycomb cysts, one of the pathologic hallmarks of IPF. In subjects with IPF, regions of dense accumulation of MUC5B were observed in areas of microscopic honeycombing and involved patchy staining of the metaplastic epithelia lining the honeycomb cysts [35].

These pathological changes are reflected in the radiological abnormalities, characteristic of IPF. Indeed, *MUC5B* polymorphism is associated with a more typical subpleural distribution of fibrosis and with a greater proportion of confident radiological diagnosis (probable UIP and UIP) [36]^.^ In our cohort, the presence of T allele *MUC5B* polymorphism was associated with a lower percentage of radiological diagnosis, which implies that carriers of the T allele did not have a CT pattern of UIP and required a histological diagnostic confirmation. In the study by Chung and coworkers [36], no information about functional parameters were given, age was lower compared to our cohort, suggesting a possible more advanced disease.

Our study has some limitation. Firstly, the study population is relatively small and there is no independent validation cohort. Secondly, the retrospective nature of the study might have introduced unintentional biases. However, the study population was carefully characterized and enrolled consecutively, which may have mitigated the selection bias. Finally, although we selected only sporadic cases, three patients were younger than 50 years, which makes one wonder about familiar disease. To the best of our knowledge they are all sporadic cases, although telomere gene mutations screening and monitoring extended to their family members would be needed to detect family aggregation.

## Conclusions

In conclusion, we have shown for the first time that MUC5B rs35705950 genotype does not seem to affect response to antifibrotic treatment in patients with IPF. In addition, carriage of the mutant T allele is associated with longer survival in IPF patients on antifibrotic treatment. Larger studies and genotyping of additional genes involved in disease pathogenesis are needed to assess the role of genotype stratification in clinical trial design and in clinical decision making.

## Supplementary Information

**Additional file 1.** Sample processing.

**Additional file 2: Table S1.** MUC5B rs35705950 genotype frequency.

**Additional file 3: Table S2.** Predictive factors of progression in the entire population of IPF patients treated with antifibrotics.

**Additional file 4: Table S3.** Occurrence of respiratory failure (RF) at rest and FVC (L) at treatment initiation according to MUC5B genotype (TT/TG vs. GG patients).

## Data Availability

The datasets used and/or analysed during the current study are available from the corresponding author on reasonable request.

## Abbreviations

IPF: Idiopathic pulmonary fibrosis
FVC: Forced vital capacity
PCR: Polymerase chain reaction
TTP: Time to progression
RF: Respiratory failure
