# Supplementary material for: Prognostic role of MUC5B rs35705950 genotype in patients with idiopathic pulmonary fibrosis (IPF) on antifibrotic treatment
Source: Respir Res. 2021 Apr 1;22:98. doi: 10.1186/s12931-021-01694-z (PMC8017848; doi:10.1186/s12931-021-01694-z)
Supplement: Supplementary file 1 — Additional file 1. Sample processing. [file 12931_2021_1694_MOESM1_ESM.docx]

**Sample processing**

All patients were genotyped for SNP rs35705950 in the promoter region of MUC5B gene by PCR amplification and Sanger sequencing. Briefly, volumes of 5 mL of blood were collected from each patient in EDTA tubes. For plasma separation samples were centrifuged at 1,800 g for 15 min within 8 h from collection. Samples of plasma and blood cell pellet were transferred to 2 mL sterile tubes, which were stored at-80°C until subsequent analyses. DNA was isolated from 300 uL of all cell pellet using the QIAamp® DNA FFPE tissue kit (Quiagen, Netherlands) in accordance with the manufacturer’s operational manual. DNA extracted from the cell pellet was used as the template for the polymerase chain reaction (PCR). PCR was performed with this primer sequence 5’-3’: forward GGTTCTGTGTGGTCTAGG, reverse: TGTTTGCTCAGCGTGTTTG. The PCR reaction phase was performed as follows:

- initial denaturation at 94°C for 5 minutes;

- three step-cycle repeated for 40 cycles: denaturation at 94°C for 15 seconds,at annealing at 56°C for 20 seconds and elongation 72°C for 20 s;

- final step maintainging the samples at 72°C for 5 min.

The amplified DNA was evaluated by agarose gel electrophoresis and its size was estimated with GeneRuler 1 kb DNA Ladder (#SM0311 -ThermoFisher scientifics, Italy). The amplified DNA was purified with PureLink® PCR Purification Kit (#K310001 - ThermoFisher scientifics, Italy) and the concentration evaluated with NanoDrop 1000 Spectrophotometer V3.8 (ThermoFisher scientifics, Italy). The purified DNA was desiccated with the forward primer and sequenced using Sanger’s technique with BigDyeTM Terminator v3.1 (ThermoFisher scientifics, Italy) on the instrument AB3730XL (ThermoFisher scientifics, Italy).

The minor allele was defined as T, and three possible combination of patient genotype could be obtained: wild type (GG), heterozygosis (TG) or variant homozygosis (TT).

Given that no genotyping method is 100% accurate and that genotype mistakes can lead to increased random error and bias in gene-disease associations, test of Hardy-Weinberg equilibrium (HWE) is widely used for a prompt check of genotype information and to detect genotyping error. This latter method is based on the assumption that in a large, randomly mating population, in the absence of disturbing forces, genotype frequencies should correspond to the HWE proportions. Deviation from these proportions can be caused by many factors, one of which is genotyping error. To validate the genotype distribution of our population, we tested our genotype frequencies for HWE.
