# Supplementary material for: Prognostic role of MUC5B rs35705950 genotype in patients with idiopathic pulmonary fibrosis (IPF) on antifibrotic treatment
Source: Respir Res. 2021 Apr 1;22:98. doi: 10.1186/s12931-021-01694-z (PMC8017848; doi:10.1186/s12931-021-01694-z)
Supplement: Supplementary file 2 — Additional file 2: Table S1. MUC5B rs35705950 genotype frequency. [file 12931_2021_1694_MOESM2_ESM.docx]

**Table S1.** MUC5B rs35705950 genotype frequency.

| **T allele:** 74/176 (42%)  **G allele:** 102/176 (58%) |  | **Observed** | **Expected** | ***p* Value** |
| --- | --- | --- | --- | --- |
|  | **TT genotype – n (%)**  **TG genotype – n (%)** | 13 (14)  48 (55) | 16 (17)  43 (49) | 0.69 |
|  | **GG genotype – n (%)** | 27 (31) | 30 (34) |  |

Chi square test for categorical variables was used.
