# Supplementary material for: Prognostic role of MUC5B rs35705950 genotype in patients with idiopathic pulmonary fibrosis (IPF) on antifibrotic treatment
Source: Respir Res. 2021 Apr 1;22:98. doi: 10.1186/s12931-021-01694-z (PMC8017848; doi:10.1186/s12931-021-01694-z)
Supplement: Supplementary file 3 — Additional file 3: Table S2. Predictive factors of progression in the entire population of IPF patients treated with antifibrotics. [file 12931_2021_1694_MOESM3_ESM.docx]

Table S2. Predictive factors of progression in the entire population of IPF patients treated with antifibrotics

|  | | | **Univariate analysis** | | **Multivariate analysis** | |
| --- | --- | --- | --- | --- | --- | --- |
|  | | | **HR (95% CI)** | ***p* Value** | **HR (95% CI)** | ***p* Value** |
| **Sex** | ***female***  ***male*** | | - | - | - | - |
|  |  |  | 1.39 (0.77 – 2.67) | 0.32 | - | - |
| **Age at diagnosis (*years)*** | ***< 70*** | | - | - | - | - |
|  | **≥ *70*** | | 0.97 (0.58 – 1.61) | 0.91 | - | - |
| **BMI (*kg/m^2^)*** | ***< 26***  **≥ *26*)** | | -  0.96 (0.58 – 1.59) | -  0.90 | -  - | -  - |
| **Smoking history (*packyears)*** | **< 10**  **≥ *10*** | | -  0.94 (0.56 – 1.56) | -  0.81 | -  - | -  - |
| **Smoking status** | ***no***  ***current***  ***former*** | | -  0.67 (0.22 – 2.04)  1.14 (0.63 – 2.07) | -  0.49  0.64 | -  -  - | -  -  - |
| **Gastroesophageal reflux** | ***no***  ***yes*** | | -  0.85 (0.50 – 1.43) | **-**  0.54 | -  - | -  - |
| **Cardiovascular diseases** | ***no***  ***yes*** | | -  0.99 (0.57 – 1.72) | -  0.99 | -  - | -  - |
| **Metabolic syndrome** | ***no***  ***yes*** | | -  1.04 (0.62 – 1.75) | -  0.86 | -  - | -  - |
| **MUC5B rs35705950** | ***TT/TG***  ***GG*** | | -  0.96 (0.56 – 1.64) | **-**  0.88 | -  - | -  - |
| **Respiratory failure at rest (*months)*** | **≥ *26***  ***< 26*** | | -  1.92 (1.12 – 3.29) | **-**  **0.02** | -  2.36  (1.12 – 4.97) | -  **0.02** |
| **Respiratory failure on effort (*months*)** | **≥ *19***  **< 19** | | -  1.67 (1.00 – 2.78) | **-**  **0.04** | -  1.15  (0.59 – 2.23) | -  0.66 |
| **Nausea and vomiting during treatment** | ***no***  ***yes*** | | -  0.48 (0.22 – 1.02) | -  0.05 | -  - | -  - |
| **Weight loss during treatment (*Kg*)** | ***no***  ***yes*** | | -  0.78 (0.43 – 1.40) | -  0.41 | -  - | -  - |
| **Diarrhea during treatment** | ***no***  ***yes*** | | -  0.92 (0.51 – 1.65) | **-**  0.78 | -  - | -  - |
| **Increase in AST and ALT** | ***no***  ***yes*** | | -  2.64 (0.35 – 19.65) | -  0.34 | -  - | -  - |
| **FVC at treatment initiation (L)** | **≥ *2.60***  ***< 2.60*** | | -  1.49 (0.90 – 2.48) | **-**  0.12 | -  - | **-**  **-** |
| **FVC at treatment initiation (%)** | **≥ *77***  ***< 77*** | | -  1.29 (0.78 – 2.14) | -  0.32 | -  - | -  - |
| **TLC at treatment initiation (%)** |  | **≥ *73***  ***< 73*** | -  1.15 (0.69 – 1.91) | -  0.57 | -  - | -  - |
| **DL_CO_ at treatment initiation (%)** | **≥ *56***  ***< 56*** | | -  1.42 (0.86 – 2.36) | -  0.16 | -  - | -  - |
| **White blood cells (n*10^9^/L)** | ***< 7.47***  **≥ *7.47*** | | -  1.45 (0.82 – 2.54) | -  0.19 | -  - | -  - |
| **Neutrophils (n*10^9^/L)** | ***< 4.18***  **≥ *4.18*** | | -  1.67 (0.95 – 2.94) | -  0.07 | -  - | -  - |
| **Neutrophils (%)** | ***< 58***  **≥ *58*** | | -  1.78 (1.00 – 3.17) | -  **0.04** | -  1.79 (0.95 – 3.03) | -  0.07 |
| **Lymphocytes (n*10^9^/L)** | ***< 2.3***  **≥ *2.3*** | | -  0.90 (0.51 – 1.57) | -  0.71 | -  - | -  - |
| **Lymphocytes (%)** | ***< 30***  **≥ *30*** | | -  0.59 (0.33 – 1.05) | -  0.07 | -  - | -  - |
| **Monocytes (n*10^9^/L)** | ***< 0.69***  **≥ *0.69*** | | -  1.23 (0.70 – 2.14) | -  0.46 | -  - | -  - |
| **Monocytes (%)** | ***< 8.5***  **≥ *8.5*** | | -  0.97(0.53 – 1.77) | -  0.93 | -  - | -  - |

FVC=forced vital capacity, TLC=total lung capacity, DLCO=lung diffusion carbon oxide, RF=respiratory failure, AST = aspartate aminotransferase; ALT = alanine aminostransferase. Values are expressed as HR (95%CI). Univariate and multivariate Cox proportional hazard regression tests were used to determine the relationship of clinical, functional and radiological characteristics with progression.
