# Supplementary material for: Prognostic role of MUC5B rs35705950 genotype in patients with idiopathic pulmonary fibrosis (IPF) on antifibrotic treatment
Source: Respir Res. 2021 Apr 1;22:98. doi: 10.1186/s12931-021-01694-z (PMC8017848; doi:10.1186/s12931-021-01694-z)
Supplement: Supplementary file 4 — Additional file 4: Table S3. Occurrence of respiratory failure (RF) at rest and FVC (L) at treatment initiation according to MUC5B genotype (TT/TG vs. GG patients). [file 12931_2021_1694_MOESM4_ESM.docx]

Table S4. Occurrence of respiratory failure (RF) at rest and FVC (L) at treatment initiation according to MUC5B genotype (TT/TG vs. GG patients).

|  | **TT/TG genotype**  **(n = 61)** | **GG**  **genotype**  **(n =27)** | ***p* Value** |
| --- | --- | --- | --- |
| **RF at rest ≥ 26 moths *– n (%)***  **RF at rest < 26 months – *n (%)***  **FVC (L) T0** **≥ 2.60 L – n (%)**  **FVC (L) < 2.60 L – n (%)** | 34 (56)  27 (44)  36 (59)  25 (41) | 11 (41)  16 (59)  9 (33)  18 (67) | 0.19  **0.02** |

Chi square test for categorical variables was used.
